# A qualitative comparison of healthcare practitioners’ perceptions regarding fatigue as a symptom in neurological conditions: insights from a tertiary care center in Saudi Arabia

**DOI:** 10.3389/fresc.2025.1433276

**Published:** 2025-05-19

**Authors:** Madawi Alotaibi, Murali P. Subramanian, Wafa Alahmari, Samiah Alqabbani, Maha Algabbani, Afrah Almuwais

**Affiliations:** ^1^ Department of Rehabilitation Sciences, College of Health and Rehabilitation Sciences, Princess Nourah Bint Abdulrahman University, Riyadh, Saudi Arabia; ^2^ Population Health Sciences Institute, Faculty of Medical Sciences, Newcastle University, Newcastle upon Tyne, United Kingdom; ^3^ Department of Rehabilitation Health Sciences, College of Applied Medical Sciences, King Saud University, Riyadh, Saudi Arabia

**Keywords:** fatigue, stroke, neurological, perception, Saudi Arabia, healthcare practitioners

## Abstract

**Introduction:**

Fatigue as a symptom severely affects individuals with neurological diseases, but its understanding and management remain limited, especially in the unique healthcare context of Saudi Arabia. While it has been extensively studied in stroke, its manifestations in neurological conditions and the perceptions of health professionals have not been thoroughly explored in this specific setting. Given the profound impact of fatigue on patient outcomes, understanding the attitudes of health professionals is crucial to identifying targeted interventions and significantly improving patient care in Saudi Arabia.

**Methods:**

This qualitative study, conducted in collaboration with a diverse group of Saudi healthcare professionals, used Semi-structured interviews to explore their perspectives on fatigue in neurological diseases. The 24 participants included physiatrists, occupational therapists, and physiotherapists, all of whom played a crucial role in treating and managing individuals with neurological conditions. Semi-structured interviews were conducted in Arabic and translated into English. Data analysis involved thematic analysis using NVivo software, with themes identified iteratively until data saturation was achieved.

**Results:**

Three overarching themes emerged from the data analysis. (1) “Fatigue is a common symptom of a neurological disorder” highlights clinicians’ recognition of the prevalence and manifestations of fatigue in various neurological disorders. (2) “Adaptive fatigue management” emphasizes the importance of individual strategies for managing fatigue, including improving endurance and environmental adaptation, and emphasizes the relationship. (3) “Fatigue and Other Conditions” explored the complex relationship between fatigue and other common neurological conditions, such as multiple sclerosis and traumatic brain injury.

**Conclusion:**

The study provides useful information about Saudi healthcare professionals’ perceptions of fatigue in neurological illnesses. Key findings stress the significance of fatigue and the need for unique designs to manage its diverse forms. While admitting limitations such as limited generalizability and inherent biases, the study advances our understanding of fatigue management in Saudi Arabia. It advocates the development of targeted therapies to improve patient care and outcomes in neurological illnesses.

## Introduction

Fatigue is a prevalent and debilitating symptom experienced by individuals with various neurological illnesses, significantly affecting their daily functioning and overall quality of life. Conditions such as multiple sclerosis (MS), Parkinson's disease, and stroke are particularly associated with fatigue. Recent research underscores the complexity of fatigue management, with cultural factors playing a crucial role. For example, a study by Induruwa et al. highlighted the neurological mechanisms underlying fatigue in MS, while Jacobi et al. pointed out the inconsistencies in guidance for post-stroke fatigue across different healthcare systems (1, 2). In Saudi Arabia, research conducted by Alahmari et al. identified both cultural and systemic barriers to effective fatigue management through patient interviews, yet the perspectives of clinicians regarding multiple neurological conditions remain largely unexplored (3).

Fatigue involves both peripheral factors, such as decreased muscle activation, and central processes, including dysfunction in brain regions like the thalamus and basal ganglia (4, 5). The impact of fatigue on patients’ daily routines can lead to early retirement and a marked decline in quality of life (6). Research shows that a significant number of individuals with neurological disorders report fatigue, with prevalence rates varying by condition. Studies indicate that 29%–77% of stroke survivors, 18%–75% of traumatic brain injury (TBI) patients, and 47%–97% of individuals with brain tumors experience fatigue (7). In movement disorders, including Parkinson's disease and dystonia, fatigue is recognized as a key non-motor symptom and is influenced by factors such as apathy, depression, and sleep disturbances (8). Similarly, patients with MS frequently report fatigue, which is closely associated with comorbidities like depression and anxiety, as well as the progression of neurological and physical impairments (9).

Despite the widespread occurrence of fatigue and its profound impact, it remains underrecognized due to the absence of a standardized definition and reliable diagnostic methods. As a result, effective treatment options are limited. The burden of neurological diseases is significant in Saudi Arabia, emphasizing the need to understand healthcare practitioners’ attitudes toward fatigue in common neurological conditions to develop effective management strategies (10, 11). These attitudes can substantially influence patient care and treatment approaches, ultimately affecting patient outcomes. Research indicates that healthcare practitioners have varying attitudes towards fatigue, ranging from recognition to skepticism (3, 12). For instance, focus groups conducted with stroke survivors in the United States revealed that some clinicians categorize fatigue as a “normal” post-stroke symptom, leading to increased patient distress (12).

Additionally, a study by Alahmari et al. explored the experiences of Saudi stroke survivors, highlighting cultural and systemic deficiencies in fatigue management, including limited clinician training and inadequate patient education (3). While these studies offer valuable insights into practitioner attitudes and patient experiences, they primarily focus on stroke and do not address fatigue across a broader spectrum of neurological disorders or consider the multidisciplinary perspectives of clinicians in Saudi Arabia. These attitudes can significantly impact patient willingness to seek help, adherence to treatment, and overall satisfaction with care. Therefore, understanding the specific attitudes of healthcare practitioners in Saudi Arabia regarding fatigue in various neurological conditions is essential for further exploration.

Unlike previous studies, which primarily concentrated on patient experiences or specific conditions like stroke, our research aims to investigate the perceptions of multidisciplinary practitioners concerning fatigue across a range of neurological disorders, including MS, TBI, and stroke, within the Saudi healthcare system. By incorporating insights from physiatrists, therapists, and occupational therapists, we identify systemic patterns in the recognition and management of fatigue, ultimately providing actionable strategies for culturally tailored interventions.

## Materials and methods

### Design

This study used qualitative research methods to help bridge the gap between clinical practice and scientific evidence by exploring participants’ beliefs, attitudes, and preferences. Personal experiences have been shown to strongly influence clinical practice and decision-making compared to scientific publications and theoretical statements (13). Purposive sampling, a non-random method of participant recruitment, was chosen for the purposes of this study. Maximum variation is the most frequently used purposeful sampling technique (14). In this method, a broad range of individuals, institutions, or settings are purposively chosen so that a wide range of participants or settings can be explored (15).

### Settings and participants

The study was carried out at the neurological rehabilitation unit of King Fahad Medical City (KFMC), a tertiary care facility in Riyadh, Saudi Arabia. KFMC is considered the first rehabilitation-focused hospital under the Ministry of Health in Saudi Arabia, with a comprehensive multidisciplinary team that includes physiatrists, physiotherapists, occupational therapists, and other rehabilitation professionals. This integration supports holistic and patient-centered care.

This neurological rehabilitation unit provides both inpatient and outpatient therapy. The inpatient neurological rehabilitation facility may handle both acute and chronic neurological conditions. Stroke, MS, TBI, and other neurological diseases are among the conditions that are frequently treated. In order to ensure continuity of care, patients frequently continue their therapy in the outpatient clinics after being discharged.

In this study, practitioners from the three primary groups (physiatrists, physiotherapists, and occupational therapists) working at KFMC who encounter fatigue in their daily practice were recruited using purposive sampling. Various studies recommend six to ten participants for individual interviews (16). However, it is common practice in qualitative research to stop conducting interviews with new participants when no additional new themes emerge – so called data saturation (17).

These practitioners commonly manage individuals with various neurological conditions, such as stroke, MS, and Parkinson's, and encounter fatigue on a daily basis. The professionals worked within the same multidisciplinary team, ensuring regular interprofessional communication and collaboration in the care of neurological patients. Individual experiences were also investigated to capture any diversity, even if their viewpoints reflect this common practice setting.

### Eligibility criteria

The following eligibility criteria were designated for the participants in this study: works as a physiatrist (doctors specializing in physical medicine and rehabilitation), physiotherapist, or occupational therapist. Must have a minimum of one year of experience in providing rehabilitation. Males and females in equal numbers from each group of practitioners.

### Data collection

The researcher (WA) met with the director of the rehabilitation unit of KFMC and explained to him the purpose of the study and the study participant inclusion criteria. A mutually convenient date and venue for the interview was arranged. Prior to the start of the interviews, the director of the department spoke to the physiatrists, physiotherapists, and occupational therapists in the department who met the study's participant inclusion criteria and verbally relayed to WA their initial consent to participate in the study. The researcher was provided with a list of the names and phone numbers of those who had expressed interest in participating in the study and contacted them to discuss their participation further. An informed consent form was given to the potential participants. Prior to the start of the interview, participants were asked to read the information sheet and sign the informed consent form.

### Interview

During the interviews, a semi-structured interview topic guide was used (Supplementary Table S1) to facilitate discussion. These questions were developed based on the findings from the broader literature review and on the basis of the aims and objectives of the study. Throughout the interviews, participants were encouraged to share their thoughts freely, with prompts for deeper understanding and clarification, while care was taken to avoid presumptions of language or bias during the discussions. In addition to the key information collected using the interview guide, other data, such as the participant's sex, qualification, occupational status, type, and years of experience, were also collected. The interviews were held in a quiet and comfortable meeting room on the KFMC. All the interviews lasted between 30 and 60 min.

### Ethical considerations

Throughout the study, participants’ confidentiality and anonymity were given the highest priority. To ensure that participants felt comfortable discussing their experiences, all interviews were held in private settings. Transcripts and audio recordings were anonymized by eliminating any identifying information, including names, job titles, and specific references from the workplace. For reporting purposes, each participant was given a unique identification number (for example, “PT1” for a physiotherapist).

Physical documents, including consent forms, were secured in a secure cabinet, and data were safely kept in encrypted files that were only accessible by the research team. During the consent procedure, participants were made aware of these procedures and given the assurance that their information would only be used for research. The King Fahad Medical City Institutional Review Board granted ethical approval (IRB No. 00,010,471).

### Data analysis

Audio recordings were transcribed verbatim, which the research team translated into English. Anonymized transcripts were imported into the NVivo (Version 14) qualitative analysis software tool. Analysis of the interview transcripts was conducted simultaneously while further interviews were conducted. No pre-determined sample size was set. However, it was envisaged that a minimum of 20 participants would be recruited, as this number is commonly recommended for sufficient information power in studies following the semi-structural qualitative interview study. Participant recruitment continued till data saturation was achieved, and no new themes emerged from the analyzed transcripts.

## Results

A study involving 24 participants was conducted, all of whom worked in KFMC's rehabilitation unit. The participants were divided equally among three groups: physiotherapists, occupational therapists, and physiatrists. Interviews were conducted for 30–60 min, and the participants’ characteristics are listed in Supplementary Table S2. Among the 24 participants, 13 were female, and 11 were male, with experience ranging from 2 to 20 years. Inductive thematic analysis was performed on the interview transcripts following Braun and Clarke (18).

The study identified three overarching themes: “Fatigue is a Neurological symptom”, which emphasizes the neurological basis of fatigue across various disorders; “Tailored Fatigue Management”, which addresses strategies for managing fatigue tailored to different neurological conditions; and “Fatigue and Other Conditions”, which investigates the relationship between fatigue and other neurological conditions such as MS and TBI.

### Theme 1 – fatigue is a neurological symptom

Fatigue, often perceived as a universal symptom, manifests uniquely within the diverse range of neurological disorders, highlighting its neurological origin in different conditions.

#### Fatigue is a symptom with of a neurological disorder

The spectrum of conditions affected by neurological fatigue was broad, as participants referenced,

… Guillain-Barré syndrome (GBS), MS, oncology patient, all of these conditions… (OT).

According to the participants’ perspective, based on their clinical experience, post-stroke fatigue is frequently less severe than fatigue in several other neurological diseases.… *Other neurological disorders from my experience, I expect that they will have fatigue more than the stroke.* (OT)

This sentiment was echoed by another, who remarked,

…because it is a continuous disease, it is a daily disease. (OT)

This daily fluctuation was further elucidated, with one participant highlighting,

The fatigue will be up and down, and daily, you will find surprises. (OT)

Notably, multiple sclerosis emerged as a prominent example, with participants recognizing its characteristic fatigability and delayed endurance improvement.

MS is known, of course, for its fatigability and symptoms, and they are among the conditions in which the improvement of endurance is delayed. (PT)

Participants highlighted that fatigue is not just physical exhaustion; it also affects cognitive functioning. One occupational therapist observed,

…. With MS patients, you notice that they struggle to focus during sessions—it’s as if they are mentally drained before they even feel physically tired… (OT)

Additionally, another physical therapist described fatigue related to TBI as

… attention deficits that accumulate throughout the day, making even simple tasks feel overwhelming… (PT)

#### Various neurological disorders

Participants expanded the discussion to encompass the multitude of neurological disorders in which fatigue plays a pivotal role. Participants acknowledged the prevalence of fatigue as a primary concern across different neurological conditions.

There are a lot of neurological disorders that fatigue can be a very primary issue. (OT)

From MS to ALS, *GBS*, and brain tumors or TBI, the range of disorders with symptoms of fatigue was extensive. The dominant nature of this pattern was attributed by participants to the significant influence of neurological illnesses on the movement and functionality of patients.

Fatigue is very common with neurological disorder… Because neurological disorder will affect the movement and the function of the patients. (PT)

These factors, combined with reduced endurance, were identified as major contributors to the widespread experience of fatigue among people living with neurological conditions by the healthcare practitioners in the study.

### Theme 2 – tailored fatigue management

This theme discusses participants’ views on understanding the complex environment of fatigue management in neurological disorders, which necessitates a tailored approach that recognizes each condition's unique needs and challenges.

#### Managing fatigue

Healthcare practitioners in the study emphasized the central objective of maintaining endurance among patients grappling with neurological disorders. One participant suggested improving the endurance of such patients.

The main goal with these patients is to increase their endurance. (OT)

Tailoring interventions to accommodate varying endurance levels was deemed essential, considering differing conditions. Another practitioner suggested:

… the main thing is that they have low endurance, but you have to accommodate it, and you cannot push them, noted a practitioner. (OT)

In contrast, stroke patients were perceived to have greater functional and physical capacity, allowing for a more assertive strategy for fatigue management. However, the challenge related to managing fatigue was acknowledged, with one participant mentioning it.

When you say I feel tired, stressed, I want to rest, I am tired of exercising, it means a lot of moving around. Plus, this is a lot of struggle. (PT)

#### Environmental modification

The importance of environmental adaptations to mitigate fatigue was mentioned, with practitioners suggesting adjustments tailored to individual needs.

You can move the furniture around, making it easier for him to perform some activities. (OT)

Similarly, for tasks like cooking, participants highlighted the significance of arranging the environment to optimize accessibility and ease of use.

#### Managing fatigue in various neurological disorders

Practitioners take a patient-centered approach, prioritizing individuals’ goals and activities in their daily lives, including home, work, and school environments. One participant explains the importance of matching interventions to patients’ individual goals and responsibilities.

Trying to prioritize the patient’s goals and activities at home, work, or school. (OT)

Meticulous planning is essential for effective fatigue management because it conserves energy reserves. A practitioner discussed the importance of proactive strategizing to maximize energy expenditure while completing necessary tasks and activities.

Planning is a vital process in energy conservation. (OT)

In order to enable patients to manage the course of their illness and the fatigue that comes with it, it is essential to create adequate awareness and appropriate education for practitioners and patients. The importance of providing patients with information about the course of their illness is highlighted by a participant comment, which enables proactive management of fatigue-related issues and informed decision-making.

… education and awareness of the illness itself… help with the management. (OT)

Since fatigue is a common symptom of many neurological illnesses, practitioners support specialized approaches to lessen its effects. A quote from one of the participants

Fatigue is very common with neurological disorders; why? Because neurological disorders will affect the movement and the function of the patients. (PT)

accentuates the intrinsic link between neurological pathology and fatigue, necessitating targeted interventions to address its manifestations.

#### Dealing with fatigue due to other conditions

In addressing fatigue stemming from other neurological conditions, practitioners advocated for multifaceted interventions encompassing education, task modification, and strategic rest periods. One participant explained,

We deal with them, as I mentioned to you, education and work simplifications and task modification. We do all of these things. (OT)

Participants also highlighted effective management strategies, such as gradually initiating interventions to build patients’ participation levels and advocating for multiple breaks to prevent cognitive overload as essential strategies for mitigating fatigue.

We give them a lot of rest periods, we start to initially gradually just to build up their level of participation. (OT)

### Theme 3 – fatigue and other conditions

This theme explores the complex relationship between fatigue and other neurological conditions, such as MS and traumatic brain injury, and stroke, throwing light on the unique manifestations and challenges encountered within these contexts.

#### Fatigue in MS patients

Participants highlighted the pervasive nature of fatigue among individuals with multiple sclerosis, acknowledging it as a prevalent and debilitating symptom. One participant mentioned highlighting the pervasive nature of fatigue within the MS population.

For neurological diseases, most of the patients with multiple sclerosis disease they are complaining of fatigue. (DR)

Environmental factors, such as hot weather, were identified as exacerbating fatigue, while interventions aimed at combating deconditioning were deemed essential in managing fatigue progression among MS patients.

We know if it is hot weather it’s exacerbates their fatigue. (DR)

#### Fatigue comparison (stroke vs. MS)

Participants delineated between fatigue experiences in stroke and MS populations, noting variations in severity and progression. While post-stroke fatigue was often observed in patients with new diagnoses, MS-related fatigue was characterized by its chronic and progressive nature. A participant explained:

MS patients eventually cannot. You see, because their muscles don't cooperate. It’s just a different level of fatigue. (OT)

#### Significant fatigue in MS

The significant impact of fatigue on individuals with MS was underscored, with participants noting its profound implications for physical and cognitive functioning. One participant observed, reiterating its debilitating effects on daily activities and quality of life.

…You can see the fatigue in a significant level with MS … but not with these two conditions. (OT)

The prominence of physical fatigue and the constant complaint of persistent tiredness among MS patients were highlighted as distinguishing features of MS-related fatigue.

It is more of physical fatigue; It’s normal because they're just trying to do things, and they say, “I'm tired”. (OT)

#### Comparison of fatigue severity

Participants compared the severity of fatigue across different neurological conditions, identifying MS-related fatigue as particularly concerning due to its constant and debilitating nature. One healthcare practitioner acknowledged the significant impact of fatigue on patients’ activity levels and overall quality of life.

In terms of severity, I think with some other diagnoses like MS, it’s more a matter of concern. (OT)

With MS, fatigue can be the factor that determines the patient’s level of activity and quality of life as well. (OT)

#### Fatigue in traumatic brain injury

While less commonly discussed among the participants in the study, fatigue in TBI was also recognized as a significant issue, often manifesting through cognitive and behavioral changes.

The fatigue itself… I don't know if it’s included there, like their behavior is agitated sometimes. (OT)

Participants highlighted the challenges of managing fatigue within the TBI population, with interventions focusing on cognitive rehabilitation and addressing agitation as potential strategies for managing fatigue.

## Discussion

This qualitative study aimed to investigate Saudi medical professionals’ perspectives on fatigue across multiple neurological conditions, including MS, TBI, and stroke. Three main themes surfaced from semi-structured interviews with 24 participants, which included occupational therapists, physiotherapists, and physiatrists: (1) “Fatigue is a symptom in Neurological disorder”, emphasizing the neurological basis of fatigue across various disorders; (2) “Tailored Fatigue Management”, which highlighted the need for personalized approaches to managing fatigue tailored to different neurological conditions; and (3) “Fatigue and Other Conditions”, which delved into the complex relationship between fatigue and other neurological conditions such as MS and TBI. The participants in the study acknowledged that fatigue was a prevalent symptom across a range of neurological illnesses, with a focus on the extent to which it impacts patients with MS and how it worsens with time. Enhancing endurance, modifying the environment, changing the way tasks are carried out, and tailored education were all strategies for dealing with fatigue related to neurological conditions. The difficulties in treating fatigue following a TBI were also recognized by practitioners, who emphasized the significance of cognitive rehabilitation and dealing with behavioral modifications.

The first theme, “**Fatigue is a symptom in Neurological disorder**” captured healthcare practitioners’ ability to recognize fatigue as a prevalent condition across all various neurological disorders. This acknowledgment aligns with existing literature, consistently identifying fatigue as a common and debilitating symptom in conditions such as MS, Parkinson's disease, and stroke (1, 19). In their review, Induruwa et al. emphasize the neurological basis of fatigue, highlighting its presence across multiple neurological conditions (1). Additionally, participants in our study highlighted fatigue's unique manifestations within different neurological disorders, indicating a nuanced understanding beyond its general characterization. This finding resonates with research on MS, where fatigue is acknowledged as multifaceted, varying across individuals and disease stages (20). Furthermore, participants recognized fatigue's fluctuating nature, aligning with research indicating daily variations influenced by temperature, stress, and physical activity, particularly evident in MS (21).

In the subsequent theme, “**Tailored Fatigue Management**”, healthcare practitioners’ perspectives on personalized approaches to managing fatigue in neurological disorders were presented. The comments by healthcare professionals are similar to those in the literature that emphasize individualized interventions for fatigue management in neurological conditions (2, 22). Jacobi et al. have also found that healthcare professionals highlighted the need to tailor fatigue guidance for post-stroke individuals (2). Participants highlighted the central objective of enhancing endurance among patients, echoing research, including systematic reviews suggesting that endurance training and physical activity can alleviate fatigue in conditions like MS and stroke (23, 24). For instance, Heine et al. found exercise interventions significantly reduced fatigue in individuals with MS, with endurance training showing particular efficacy (23). Within this theme, healthcare professionals also emphasized the importance of environmental modifications to mitigate fatigue, aligning with studies suggesting that such adaptations can conserve energy and reduce fatigue in individuals with neurological disabilities (25). Participants in the study also discussed tailoring interventions to accommodate varying endurance levels across different neurological conditions, which has been highlighted in the literature as a successful strategy in fatigue management. For example, Kalron et al. demonstrated that tailored rehabilitation programs improved endurance and reduced fatigue in individuals with MS, emphasizing the significance of personalized interventions (26). Furthermore, practitioners in this study emphasized the importance of comprehensive interventions that combine education, task modifications, and strategically planned rest periods to effectively address individual needs. Similarly, Jacobi et al. highlighted the critical role of providing timely education about fatigue to individuals recovering from stroke (2). Additionally, healthcare providers in their study advocated for the importance of pacing exercises as a key strategy to better accommodate the unique needs of each participant.

The final theme, “**Fatigue and Other Conditions**”, explores the complex relationship between fatigue and other neurological conditions like MS and TBI. The prevalent nature of fatigue in MS was emphasized by participants, aligning with existing research that highlights its high prevalence and negative impact on quality of life (1, 19, 27). Differences in severity and progression of fatigue between MS and stroke have been extensively researched. The participants included in our study also discussed these variations in fatigue experiences between stroke and MS populations (28, 29). Additionally, participants highlighted the significant impact of fatigue on physical and cognitive functioning in individuals with MS. Fatigue in MS patients impairs cognitive function, physical activity, and social participation, contributing to decreased overall well-being (20, 30). Wylie et al. stressed the significance of addressing cognitive and behavioral aspects in TBI-related fatigue management (31), while participants in our study also highlighted the challenges in the management of fatigue in the TBI population owing to its complex nature that includes physical, cognitive, and emotional elements.

## Limitations

This study provides valuable insights into managing fatigue as a symptom of common neurological diseases among a multidisciplinary team in a tertiary care center. However, it is important to consider some limitations of the study. Firstly, the study's participants were limited to individuals with experience managing fatigue as a symptom of neurological diseases and working in a single tertiary care setting. This focus may have homogenized participant views and limited the generalizability of the findings to less integrated and different levels of healthcare settings. Hence, the conclusions may not accurately represent the opinions of all Saudi medical professionals.

The study also did not explore possible cultural implications on attitudes toward fatigue, which limits our understanding of how cultural factors may influence the perceptions of Saudi Arabian healthcare practitioners.

The integrated multidisciplinary care approach at KFMC supports holistic care to managing fatigue but may limit variability in professional perspectives, potentially confounding findings. Future studies might explore less integrated settings, different care environments in Saudi Arabia, and certain professional subgroups in order to overcome these limitations. Quantitative research might further assess how multidisciplinary care affects fatigue-related outcomes, providing additional knowledge on the benefits of integrated care approaches.

## Conclusion

This study offers valuable insights into Saudi healthcare professionals’ perspectives regarding fatigue in common neurological conditions. The main conclusions highlight fatigue's status as a common and complex symptom in various neurological conditions, focusing on its neurological origins and its significant effects on everyday functioning and patients’ quality of life. The study further highlights the significance of customized approaches to fatigue management, such as methods to increase endurance, changes to the environment, and interventions customized for various neurological diseases. The complex links between fatigue and other common neurological disorders like TBI and MS highlight the necessity of thorough evaluations and focused interventions to address the range of symptoms and difficulties related to fatigue in these populations. This study clarifies these attitudes and challenges, which advances the understanding of fatigue management within the Saudi healthcare system and guides the development of focused interventions meant to enhance patient care and outcomes for those suffering from neurological disorders.

## Data Availability

The original contributions presented in the study are included in the article/Supplementary Material, further inquiries can be directed to the corresponding author.
