# Supplementary material for: A qualitative comparison of healthcare practitioners’ perceptions regarding fatigue as a symptom in neurological conditions: insights from a tertiary care center in Saudi Arabia
Source: Front Rehabil Sci. 2025 May 19;6:1433276. doi: 10.3389/fresc.2025.1433276 (PMC12127392; doi:10.3389/fresc.2025.1433276)
Supplement: Supplementary file 1 [file Table1.docx]

**Supplement**

**Table 1: The** **Semi structure interview topic guide**

| **Q#** | | **Topic guide** |
| --- | --- | --- |
| 1 | In your work, have you observed fatigue in any particular neurological disorders?" | |
| 2 | Can you share any cases where fatigue was prominent in a neurological patient? | |
| 3 | How do you deal with managing fatigue in patients with different neurological conditions?" | |
| 4 | Can you share some examples of how you tailored fatigue management based on the underlying neurological disorder? | |
| 5 | What treatment options have you found work in managing fatigue in different neurological cases? | |
| 6 | Is there anything else you'd like to share about your experience with fatigue in neurological patients that we haven't talk about it? | |

Table 2: Characteristics of the study participants

| **Unique ID** | **Sex** | **Highest educational attainment** | **Occupation status** | **Occupation level** |
| --- | --- | --- | --- | --- |
| PT1 | F | BSc | Full time | Senior PT |
| PT2 | M | MSc | Full time | Senior PT |
| PT3 | F | BSc | Full time | Senior PT |
| PT4 | M | MSc | Full time | Senior PT |
| PT5 | F | MSc | Full time | Senior PT |
| PT6 | F | MSc | Full time | Senior PT |
| PT7 | F | MSc | Full time | Senior PT |
| PT8 | M | BSc | Full time | PT |
| OT1 | M | BSc | Full time | Senior OT |
| OT2 | F | BSc | Full time | OT |
| OT3 | M | BSc | Full time | Senior OT |
| OT4 | M | BSc | Full time | OT |
| OT5 | M | BSc | Full time | OT |
| OT6 | F | BSc | Full time | OT |
| OT7 | F | BSc | Full time | Senior OT |
| OT8 | F | BSc | Full time | Senior OT |
| DR1 | M | Doctorate | Full time | Associate consultant |
| DR2 | F | Doctorate | Full time | Consultant |
| DR3 | M | Postgraduate diploma | Full time | Assistant consultant |
| DR4 | F | Postgraduate diploma | Full time | Assistant consultant |
| DR5 | F | Postgraduate diploma | Full time | Assistant consultant |
| DR6 | M | Postgraduate diploma | Full time | Assistant consultant |
| DR7 | F | BSc | Full time | Resident |
| DR8 | M | BSc | Full time | Resident |

Notes: M = male; F = female; BSc = Bachelor of Science; MSc = Master of Science; PT = physical therapist; OT = occupational therapist; DR = physiatrist.
